# Supplementary material for: AbTune: layer-wise selective fine-tuning of protein language models for antibodies
Source: Brief Bioinform. 2026 Jul 12;27(4):bbag374. doi: 10.1093/bib/bbag374 (PMC13356902; doi:10.1093/bib/bbag374)
Supplement: AbTune_SI_bbag374 [file abtune_si_bbag374.pdf]

Supplementary material

AbTune: layer-wise selective fine-tuning of protein language models for antibodies

Xiaotong Xu[1*], Alexandre MJJ Bonvin[1*]
[1]Computational Structural Biology Group, Bijvoet Centre for Biomolecular Research, Department of Chemistry, Faculty of Science, Utrecht, Netherlands
*To whom correspondence should be addressed
Emails: x.xu1@uu.nl a.m.j.j.bonvin@uu.nl

SI Figure 1. Distribution of the number of data points per antibody-antigen complex for application 2 2

SI Figure 2. The correlation between Perplexity and best performing step in application 2 2

SI Method. Training and model details of BindFormer 3

SI Table 1. Performance of different BindFormer various under different fine-tuning scenarios 3

SI Figure 1. Distribution of the number of data points per antibody-antigen complex for application 2 (mutation effect prediction)**.** Each bar represents the number of complexes with a given count of point mutation.

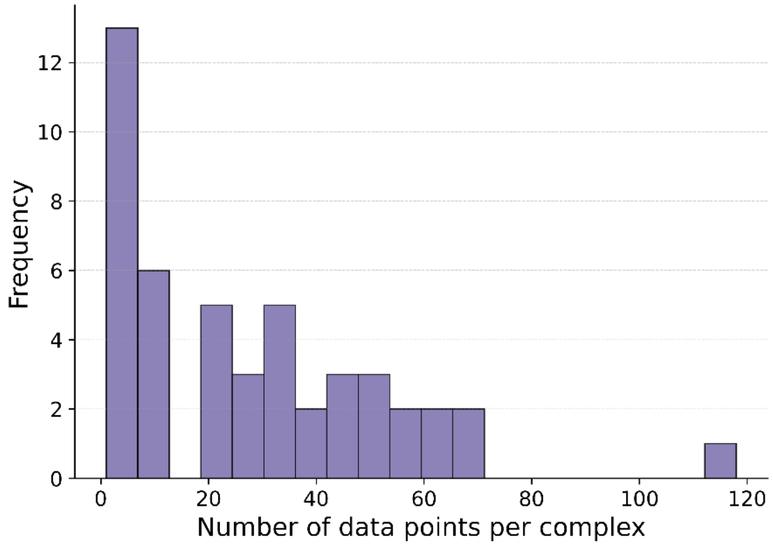

SI Figure 2. The correlation between Perplexity and best performing step in application 2 (mutation effect prediction). We report here the moderate correlation between initial perplexity and the best forming step with our best performing model ESM-t12-AbTune.

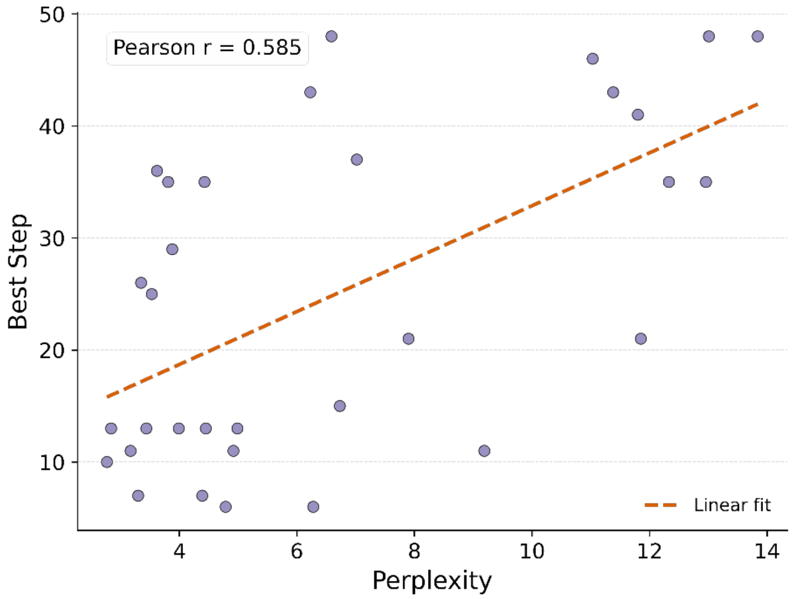

SI Method. Training and model details of BindFormer

Our BinderFormer architecture models antibody heavy and light chain sequences using a dual-chain transformer-based encoder. Each chain is first embedded with ESM-2 (with default or fine-tuned weights) and then projected to a higher-dimensional space (d_attn = 128) via a linear layer. Sequential signals within each chain are captured using Rotary Multi-Head Attention (RoPE) with 4 attention heads. The attention outputs are then refined through a two-layer SiLU MLP with residual connections. A learned attention pooling module aggregates per-residue representations into a single vector for each chain. Finally, embeddings from the heavy and light chains are concatenated and passed through a three-layer fully connected classifier with ReLU activations, Batch Normalization, and Dropout (p = 0.2) for final classification output.

SI Table 1. Performance of different BindFormer various under different fine-tuning scenarios

Model (Vary by BinderFormer scenarios)	Accuracy	F1	Precision	Recall	AUC
baseline	0.942	0.943	0.945	0.942	0.98
0.1%-top-10step	0.93	0.93	0.935	0.93	0.975
0.1%-top-50step	0.956	0.951	0.957	0.956	0.99
0.1%-rand-10step	0.951	0.952	0.953	0.951	0.982
0.1%-rand-50step	0.977	0.977	0.977	0.977	0.996
1%-top-10step	0.93	0.932	0.936	0.93	0.968
1%-top-50step	0.977	0.976	0.977	0.977	0.996
1%-rand-10step	0.906	0.878	0.855	0.906	0.789
1%-rand-50step	0.973	0.973	0.973	0.973	0.995

Each model name follows the format <Percentage>% - <Selection Method> - <Training Steps>, where the percentage indicates the fraction of sequences selected for fine-tuning, the selection method specifies whether sequences were chosen based on highest perplexity(top) or randomly (rand), and the training steps indicate how many steps the model was fine-tuned on the selected sequences; for example, 0.1%-top-10step means the top 0.1% of sequences were fine-tuned for 10 steps.
